# Circular RNAs in acute myeloid leukemia

**DOI:** 10.1186/s12943-021-01446-z

**Published:** 2021-11-18

**Authors:** Vijendra Singh, Mohammed Hafiz Uddin, Jeffrey A. Zonder, Asfar S. Azmi, Suresh Kumar Balasubramanian

**Affiliations:** 1 Department of Oncology, Karmanos Cancer Institute/Wayne State University, 4100 John R, HWCRC 740.2, Detroit, MI 48201 USA; 2 Department of Oncology, Wayne State University School of Medicine, 4100 John R, HWCRC 732, Detroit, MI 48201 USA

**Keywords:** Acute myeloid leukemia, Non-coding RNA, Circular RNA

## Abstract

Although mechanistic studies clarifying the molecular underpinnings of AML have facilitated the development of several novel targeted therapeutics, most AML patients still relapse. Thus, overcoming the inherent and acquired resistance to current therapies remains an unsolved clinical problem. While current diagnostic modalities are primarily defined by gross morphology, cytogenetics, and to an extent, by deep targeted gene sequencing, there is an ongoing demand to identify newer diagnostic, therapeutic and prognostic biomarkers for AML. Recent interest in exploring the role of circular RNA (circRNA) in elucidating AML biology and therapy resistance has been promising. This review discerns the circular RNAs’ evolving role on the same scientific premise and attempts to identify its potential in managing AML.

## Introduction

Acute myeloid leukemia (AML) is characterized by clonal expansion of myeloid blasts in the bone marrow and peripheral blood. AML was originally classified based on the morphological appearance of the myeloid blasts [1], and subsequently according to whether recurrent characteristic chromosomal abnormalities (with specific resultant gene fusions and point mutations in different myeloid genes) were present [2, 3]. As the mechanistic underpinnings associated with these genetic alterations have been elucidated, the clinical utility of detailed molecular characterization of AML has extended beyond simply providing a framework for disease classification, now permitting prognostication, response monitoring, and – increasingly – patient-specific treatment decisions. Despite these advancements, the overall prognosis remains dismal [5-year overall survival 28.7%] [4]. Inherent and acquired resistance to therapy contributes to high rates of early disease relapse and death, even in patients not initially identifies as having “high-risk” molecular features, underscoring the need for innovative, predictable, and robust alternative biomarkers.

Recent advances in high-throughput sequencing demonstrated that the majority of mRNA (~ 75%) of the entire human transcriptome do not get translated into proteins and are called non-coding RNA (ncRNA) [5]. ncRNAs are divided based on transcript sizes into small (< 200 nucleotides) and long (> 200 nucleotides) RNAs (sncRNAs and lncRNAs, respectively). While themselves not being translated into proteins, ncRNAs regulate gene expression by regulating transcription, post-transcriptional modifications, and translation [6, 7]. Altered ncRNAs may have a pathogenic role in the development of different cancers, including AML, in which they have been implicated in both leukemogenesis and drug resistance [8].

**Fig. 1:**
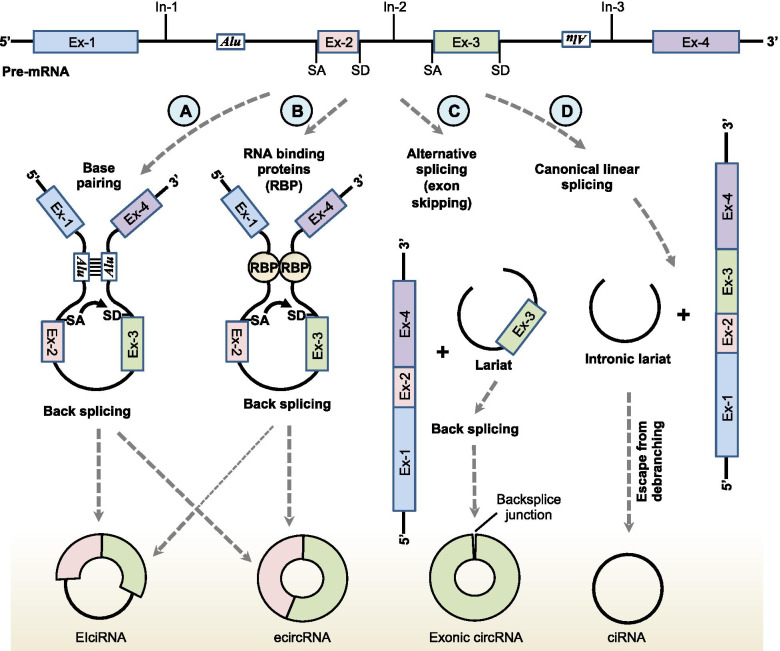
The biogenesis of diverse circRNA. There are several mechanisms of circRNA biogenesis. **A** Intron pairing mediated circularization. **B** RBP mediated circularization. Backsplicing of RNA is favored by long flanking introns, the presence of inverted repeat elements (e.g., *Alu* elements), and trans-acting RNA binding proteins (**A**, **B**) [70]. The base pairing between different introns brings together upstream splice acceptor (SA) and downstream splice donor (SD) sites, leading to backsplicing. During backsplicing, SD attacks SA, which results in exon-intron circRNAs (EIcircRNAs) or exonic circRNAs. **C** Alternative splicing mediated circularization (lariat-driven). The circRNAs also can originate from alternative splicing intermediates called lariat precursors. In this process, RNA is spliced linearly but skips specific exon. **D** Canonical linear splicing mediated circularization (intronic lariat-driven). Here intronic lariat precursors that avoid the debranching step make circRNA exclusively contain introns. Ex, exon; In, intron; RBP, RNA binding protein; SA, splice- acceptor site; SD, splice- donor site; EIcircRNAs, exon-intron circRNAs; *Alu, Alu* inverted repeat elements; BSJ, backsplice junction; ciRNA, circular intronic RNA

CircRNA is a single-stranded ncRNA which, due to a closed-loop structure secondary to a phosphodiester bond between the 3′ and 5′ ends, as well as the absence of a 3′ polyadenyl tail, has increased stability and a longer half-life compared to linear RNA [9]. The same structural uniqueness also makes them resistant to nucleases [10]. CircRNAs are produced from canonical or non-canonical splicing of any of several parts of the primary transcripts, including exon, intron, 3′ and 5′ untranslated regions, intergenic regions, and sometimes from antisense RNAs [11, 12]. Based on the primary transcript region involved in splicing, the resultant circRNA can be exonic circRNA, intronic circRNA, intron-exon circRNA, and intergenic circRNA [9, 11–13]. Most described circRNAs are of exonic subtype. Fusion circRNA has been identified in malignancies associated with chromosomal translocations [14]. Various mechanisms of circRNA biogenesis are demonstrated in Fig. 1. The majority of circRNAs are located in the cytoplasm, and the number of circRNA transcripts is enriched relative to the corresponding amount of linear RNA derived from a particular gene [15]. CircRNA expression is tissue and developmental stage-specific [13, 16]. CircRNA regulates gene expression by directly or indirectly altering transcription and translation through several mechanisms [17], including by sponging or decoying microRNA (miRNA), thereby reducing miRNA-mediated degradation of messenger RNA (mRNA) [18]. CircRNA can also directly interact with proteins and modulate their function [19]. For example, circRNA binds and decoys RNA binding proteins (RBPs) [20], and it also facilitates co-localization of enzymes by serving as a protein scaffold [21]. Though circRNAs are considered to be one type of ncRNA, recent studies have confirmed that they can be translated in some cases [22, 23].

Long non-coding RNA (lncRNA) is another ncRNA produced during transcription by RNA polymerase II and is expressed in a tissue-specific manner [24]. In contrast to circRNA, most lncRNAs are localized to the nuclear compartment. Otherwise, circRNA and lncRNA share certain common properties (i.e., being non-coding, highly conserved, and having a relatively long half-life) and functions (e.g., sponging miRNAs). Both RNA species have specific mechanisms of action relevant to pathogenesis of disease, including leukemia [25, 26].

CircRNA, specifically, has been implicated in Alzheimer’s disease, diabetes mellitus, cardiovascular diseases, and many other conditions, including but not limited to cancer [27]. Aberrant expression of circRNAs has been implicated in various steps of tumorigenesis, metastasis, and drug resistance [28]. The significance of circRNA in cellular homeostasis and tumorigenesis, especially considering its abundance in body fluids, including blood, makes it ideal as a potential prognostic and diagnostic biomarker [29–31]. This review summarizes several circRNAs with altered expression reported in AML, their role in disease biology, and their diagnostic, prognostic, and therapeutic potential.

### CircRNA as a biomarker for diagnosis and prognosis in AML

Multiple studies have explored the potential use of circRNAs in the diagnosis and prognostication of AML. Variable expression of specific circRNAs studied in different model systems have been found to be prognostic in AML. Yi et al. demonstrated upregulation of circ-VIM by means of reverse transcriptase and quantitative polymerase chain reaction (RT-qPCR) in AML patients’ bone marrow samples compared to healthy controls [32]. Based on circ-VIM levels, AML patients can be divided into two cohorts: low and high expressers. Circ-VIM expression correlated with certain clinical features, such as white blood cell (WBC) counts and French-American-British (FAB) AML morphologic subtypes. In multivariate analysis, high circ-VIM expressers showed shorter leukemia-free and overall survival. The accuracy of circ-VIM as a biomarker was demonstrated by the area under the receiver-operating characteristic curve (AUC of ROC) of 0.741 comparing AML patients to healthy controls. In this study, the expression of parental gene *VIM* positively correlated with circ-VIM expression. Though *VIM* expression’s prognostic significance was not assessed in this study, authors alluded to the possibility of circ-VIM as a tumor promoter.

Similarly, in another study, circ-Foxo3 was downregulated in de novo AML patients, and circ-Foxo3 expression positively correlated with Foxo3 gene expression [33]. ROC analysis suggests circ-Foxo3 expression may have diagnostic utility in AML. Conclusions regarding its use as prognostic factor were less clear, as high Foxo3 gene expression was associated with a better prognosis but the circ-Foxo3 expression was not.

In another study, microarray-based analysis revealed 147 up and 317 downregulated circRNAs respectively in six newly diagnosed cytogenetically normal AML (CN-AML) patients with varying prognostic risk based on *FLT3* and *NPM1* mutation status compared to four healthy controls [34]. Further, a molecular signature based on the expression pattern of 5 specific circRNAs (2 upregulated; hsa_circi_0035381 and hsa_circ_0049657, and 3 downregulated; hsa_circ_0001187, hsa_circ_0008078, and hsa_circ_0001947) which could distinguish between high and low-risk AML patients was identified. Hsa_circ_0004277, another circRNA downregulated in this cohort seems to be a promising diagnostic biomarker, with an AUC of ROC of 0.957 (AML patients versus healthy controls). More interesting perhaps was the finding that in a validation group of 107 AML patients at various stages of treatment, the significantly downregulated Hsa_circ_000427 expression observed at diagnosis was restored to control levels at remission, and then became downregulated again at disease relapse. Thus, serial measurement of hsa_circ_0004277 may potentially help in disease surveillance, as well as initial diagnosis.

Circ_009910 (exonic circRNA encoded by *Mitofusin 2* gene on chromosome 1) and circ-ANAPC7 were upregulated in AML patients compared to controls with iron deficiency [35, 36]. Circ_0009910 regulates cell cycle progression, proliferation, and apoptosis of leukemic cells by sponging miR-20a-5p. Poor-risk AML patients, particularly, had high circ_009910 levels, but the question of whether this had an impact on survival independent of the established factors used to define the poor-risk category was not evaluated.

More recently, whole-genome microarray comparing bone marrow mononuclear samples from extramedullary infiltrating (EMI) AML and non-EMI AML patients revealed 253 and 259 circRNAs to be up and downregulated, respectively, in EMI compared to non-EMI AML patients [37]. The authors identified 7 target genes regulated by 17 circRNAs (*LRRK1, PLXNB2, OLFML2A, LYPD5, APOL3, ZNF511,* and *ASB2*) associated with poor prognosis, and 2 genes (*PAPLN* and *NRXN3)* whose overexpression correlated with better prognosis.

Another study demonstrated circ-PTK2 (hsa_circ_104700/hsa_circ_0005273) to be upregulated in AML bone marrow samples compared to healthy controls [38]. Knockdown (KD) of circ-PTK2 suppressed proliferation and triggered AML cells’ apoptosis by decreasing the expression of cyclin D1 and anti-apoptotic protein BCL-2 and increasing expression of pro-apoptotic protein Bax levels. In-vivo studies demonstrated that circ-PTK2 bound and sequestered miR-330-5p, leading to increased expression of *FOXM1*. High expression of circ-PTK2 was associated with shorter survival in AML patients, presumably due to its effect on leukemic cell proliferation and apoptosis.

In another study, microarray analysis performed on bone marrow samples from patients with acute lymphoblastic leukemia (ALL) and AML revealed differential expression of 10 specific circRNAs [39]. For example, hsa_circ_0012152 was found to be upregulated in AML compared to ALL (AUC of ROC 0.8625) and healthy controls (AUC of ROC 0.9773), while hsa_circ_0001857 was upregulated in ALL vs. AML (AUC of ROC 0.90911).

In summary, circRNAs have potential utility in AML as a diagnostic and prognostic biomarker and as a means of monitoring disease status and therapeutic response. The role of circRNA measurement to monitor disease status in the context of commercially available clinical measurable disease (MRD) monitoring remains to be determined. As MRD influences AML treatment decisions, it creates more opportunities to study circRNA further in this setting.

### Role of circRNA in elucidating AML biology

**Fig. 2:**
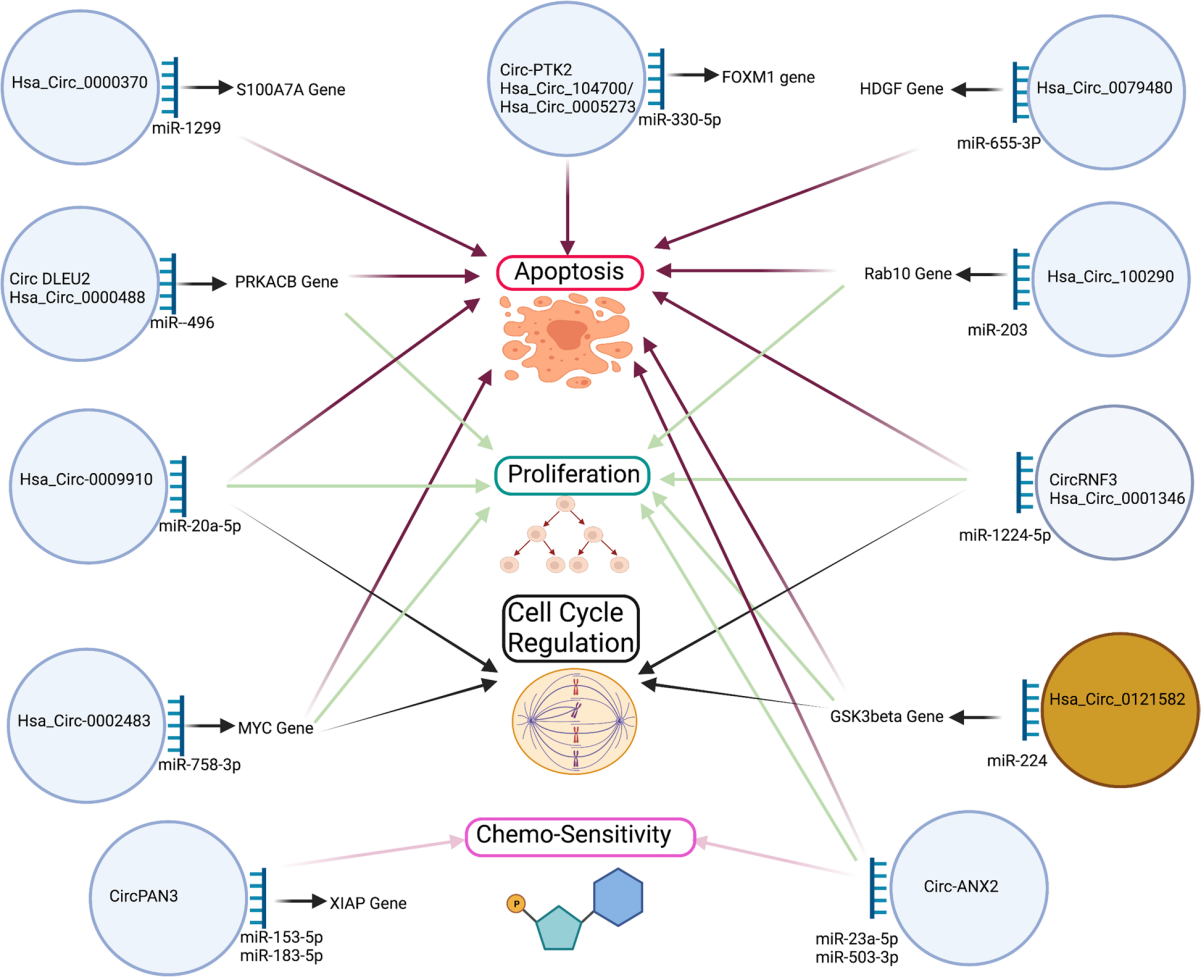
Schematic representation of the role of circRNA in AML biology. CircRNA’s roled in apoptosis, leukemic cell proliferation, regulation of cell-cycle and chemo-sensitivity are through sponging of miRNA. Light blue circle indicates upregulated circRNA in AML and yellow circle indicates downregulated circRNA in AML. Gene that is affected by circRNA mediated sponging is shown by an arrow

Apart from being a diagnostic/prognostic biomarker in AML, the role of circRNA in AML biology and pathogenesis has also been explored (Table 1 and Fig. 2). There is a growing body of evidence that circRNA regulates gene expression and modulates various steps of leukemogenesis, including differentiation, proliferation, cell cycle transition, adhesion, and apoptosis.

**Table 1:** CircRNAs in acute myeloid leukemia. ↑ Indicate increased expression, ↓ Indicate decreased expression. BM (Bone marrow). Ref. (References)

CircRNA Circ based ID (Common Name)	Host gene	Sample (Expression pattern)	Method	Target miRNA/Target gene	Function	Ref.
Circ-VIM	Vimentin	BM (↑)	qRT-PCR		Diagnostic/prognostic biomarker	[32]
Circ-FOXO3	FOXO3	BM (↓)	qRT-PCR		Diagnostic biomarker	[33]
Hsa_circ_0004277	WDR37	BM (↓)	Microarray/qRT-PCR		Diagnostic marker, dynamic marker with disease status	[34]
Circ_0009910	MFN2	BM (↑)	Microarray/qRT-PCR	miR-20a-5p	Prognostic marker	[35]
Hsa_circ_101141 (Circ-ANAPC7)	ANAPC7	BM (↑)	Microarray/qRT-PCR			[36]
Hsa_circ_104700/Hsa_circ_0005273 (Circ-PTK2)		BM (↑)	qRT/PCR	miR-330-5p/FOXM1	Prognostic marker	[37]
Hsa_circ_0100181 (CircPAN3)	PAN3	BM (↑)	Microarray/qRT-PCR	miR-153-5p, miR-183-5p/XIAP	Chemoresistance	[57, 58]
Hsa_circ_0035559 (Circ-ANXA2)	ANXA2	BM (↑)	Microarray/qRT-PCR	miR-23a-5p and miR-503-3p	Prognostic biomarker/ Chemoresistance	[59]
Hsa_circ_0000488 (Cir-DLEU2)	DLEU2	BM (↑)	Microarray	miR-496/PRKACB	Cell proliferation and inhibition of apoptosis	[41]
Circ_0000370	FLI-1	Blood (↑)	Microarray	miR-1299/S100A7A	Oncogene	[44]
Hsa_circ_0006332 (CircMYBL2)	MYBL2	(↑)		FLT3 translation regulation through PTPB1	Oncogene Therapeutic potential	[45]
Hsa_circ_0075001	NPM1	BM (↑)	Microarray		Downregulation of Toll-like receptor signaling	[54]
Hsa_circ_0004136		BM (↑)	Microarray/qRT-PCR	miR-142 and miR-29a	Cell proliferation	[48]
Hsa_circ_0001346 (circRNF13)	RNF13	Blood (↑)	qRTPCR	miR-1224-5p	Cell proliferation and migration	[49]
Hsa_circ_0121582	GSK3beta	BM (↓)	High-throughput sequencing/qRTPCR	miR-224/GSK3beta	Inhibition of proliferation in AML cells	[50]
Hsa_circ_100,290	SLC30A7	BM (↑)	qRTPCR	Mir-203/Rab10	Regulation of proliferation and apoptosis	[51]
Hsa_circ_0079480	ISPD	BM (↑)	qRT-PCR	miR-655-3p/HDGF	Regulation of proliferation and apoptosis	[53]
Hsa_circ_0002483		BM (↑)	qRT-PCR	miR-758-3p/MYC	Regulation of proliferation, cell-cycle progression, and apoptosis	[52]

### Regulation of apoptosis, cell cycle progression, and proliferation

Although there are numerous factors contributing to leukemogenesis in AML, evading apoptosis seems to have a critical role [40]. A recent study demonstrated higher expression of circ-DLEU2 (Hsa_circ_0000488) in AML patients than healthy controls [41]. Higher circ-DLEU2 expression correlated with impaired apoptosis, increased cell proliferation in vitro*,* and accelerated tumor growth in vivo. Circ-DLEU2 upregulated *PRKACB* gene expression via sponging of miRNA-496. Note that PRKACB protein is the catalytic subunit of cyclic AMP-dependent protein kinase and is thus involved in regulating various cell signaling processes. While studies have reported the role of parent gene *DLEU2* in leukemogenesis [42, 43], *DLEU2* expression and its effects were not investigated in this study.

In a study by Zhang et al., both circ_0000370 and its parent gene *FLI-1* were found to be upregulated in AML patients [44]. Circ_0000370 expression was higher in *FLT3-ITD* patients than in *FLT3* wild-type (WT) patients and was associated with a poor prognosis. In Vitro, *FLT3*-ITD expression positively correlated with circ_0000370 expression, and quizartinib (a FLT3 inhibitor) downregulated circ_0000370 expression. Circ_0000370 regulated cell survival and apoptosis via increased *S100A7A* expression by sponging miR-1299. In a different study, Sun et al. demonstrated that circMYBL2 (has_circ_0006332, parent gene *MYBL2*, a cell cycle checkpoint gene) expression to be approximately five-fold higher in *FLT3*-ITD AML patients than in *FLT3* WT patients [45]. CircMYBL2 inhibited apoptosis, enhanced proliferation, and facilitated cell-cycle progression in *FLT3*-ITD leukemic cells but not *FLT3* WT cells. Further, circMYBL2-KD led to reduced FLT3 kinase expression, resulting in modulation of downstream signaling, like reduced phosphorylation of STAT5 (which in turn resulted in reduced expression of *c-MYC*). In addition, circMYBL2 enhanced translation of FLT3 kinase by facilitating the polypyrimidine tract-binding protein 1 (PTBP1) binding to FLT3 messenger RNA. In previous studies, *FLT3*-ITD mutation was associated with increased MCL1 and decreased p27/Kip1 expression, which led to enhanced apoptosis resistance [46, 47]. CircMYBL2-KD was also associated with the reversal of these effects on MCL1 and p27/Kip1 expression levels. In vivo studies demonstrated circMYBL2-KD inhibited *FLT3-*ITD AML progression and was associated with improved survival in mice with quizartinib sensitive and resistant AML. Overall, these results suggest the effects of driver mutations like *FLT3* ITD in AML biology may be mediated (or at least modulated) by altered expression of circRNAs (circ_0000370 and circMYBL2).

In another study, 273 and 296 circRNAs were up and downregulated respectively in pediatric AML patients compared to healthy controls [48]. Circ-004136, produced from the RING finger protein 13 (*RNF13*) gene at chromosome 3q25.1, was significantly upregulated in pediatric AML patients. Circ-004136 sponges miR-142 and miR-29a and regulates AML cell proliferation. Another study also demonstrated upregulation of has_circ_0001346 (circRNF13) in AML patients [49]. In this study, circRNF13 down-regulation was associated with reduced proliferation, cell cycle arrest, increased apoptosis, attenuation of migration, and invasion of AML cells by acting as a sponge for miRNA1224-5p. These effects were mediated by c-MYC regulation at the mRNA level, Caspase 3/7 activation, and reduced Tenascin-C expression at the molecular level. Together these studies suggest circRNA exerts its leukemogenic potential via sponging different miRNAs.

Both the circRNA (hsa_circ_0121582) and its parent gene *GSK3beta* (a tumor suppressor) are shown to be down-regulated in leukemia cells [50]. Hsa_circ_0121582 is present both in the cytoplasm and in the nucleus, with different functions evident in each of these compartments: in the cytoplasm, it acts as a sponge for miR-224, and in the nucleus, it binds to the *GSK3beta* promoter to recruit DNA demethylase *TET1*. The resultant upregulation of GSK3beta expression inhibits the Wnt/beta-catenin signaling pathway, leading to leukemia cell proliferation.

Other investigators have also demonstrated an association between circRNA and evasion of apoptosis. Hsa_circ_100,290 (encoded by parent gene SLC30A7) was upregulated in AML patients [51]. Hsa_circ100290-KD in cell lines inhibited cell proliferation and accelerated apoptosis by targeting miR-203, resulting in regulation of cyclin D1, CDK4, BCL-2, and cleaved Caspase-3 expression, even though expression of host gene SLC30A7 was not affected in this knockout model. It also regulated RAB10 expression, a member of the RAS superfamily of small GTPases, similarly via targeting miR-203. Another circRNA, hsa_circ_0002483, was overexpressed in AML patients compared to iron-deficient controls [52]. Hsa_circ_0002483-KD led to decreased cell proliferation, cell cycle arrest (G0/G1 phase), and increased apoptosis via reduction of BCL-2 and elevation of BAX and C-caspase-3. It also upregulated MYC expression via sponging miR-758-3p. In another study, hsa_circ_0079480 expression was higher in AML than in idiopathic thrombocytopenia patients [53]. Hsa_circ_0079480-KD was associated with lower viability and increased apoptosis of AML cells. Hsa_circ_0079480 upregulated HDGF (heparin-binding nuclear growth factor) expression via sponging miR-655-3p. In the same study, low miR-655-3p expression and high HDGF expression were associated with poor survival in AML patients.

Whether altered expression of circRNA is sine qua non for AML leukemogenesis, or whether it is simply one of several potentially overlapping abnormalities affecting several steps in AML disease biology remains to be fully determined. Much of the work described herein suggests sponging miRNA and resultant alteration of target gene expression is a central function of circRNA as it relates to leukemogenesis, other mechanisms, including direct binding to the gene promoter regions, need to be further characterized, as well.

### The pattern of circRNA expression and AML phenotype

The historical classification of acute leukemia (FAB) relied on lineage and maturational features of blasts, as reflected by morphological features. A global evaluation of circRNA expression from healthy hematopoietic controls and AML patients identified hematopoietic differentiation-associated and AML subgroup-specific signatures [54]. Hsa_circ_0075001, a circRNA of the *NPM1* gene, followed a similar expression pattern as the parent *NPM1* gene, independent of *NPM1* mutation status. High expression of hsa_circ_0075001 associated with no or minimal blast maturation and down-regulation of Toll-like receptor signaling pathway.

Similarly, in another study, whole-transcriptome profiling of 365 younger adults with CN-AML [55] identified 3 distinct circRNA expression-based clusters. These were associated with different clinical and molecular features, e.g., differences in age, extramedullary manifestations, somatic mutations, white blood cell count, platelet count, and blast count. CircFBXW7 was identified to be a regulator of the proliferative capacity of AML blasts. High vs. low expression of circFBXW7 associated with a distinct gene expression signature. No miRNA-binding site was identified for circFBXW7 suggesting that there might be a different mechanism of action than the miRNA sponging described previously. Higher circCFLAR, circKLHL8, circSMC1A, and circFCHO2 expression were each associated with a better prognosis. In each case, except for circFCHO2, correlation with the corresponding linear RNA isoform was weak, indicating observed effects were from circRNA rather than parent gene more generally.

### CircRNA and drug resistance in AML

Primary or secondary refractoriness to treatment (i.e., inherent and acquired drug resistance) is a key limiting factor for optimal outcomes in AML patients. Genetic alterations leading to aberrant activation of drug resistance-related signal pathways is a well-known resistance mechanism in AML [56]. Elucidation of the role of ncRNAs, especially circRNAs, in AML drug resistance, is emerging, potentially providing an avenue for future research developing novel treatment strategies to overcome drug resistance.

Shang et al. studied circRNA expression patterns in doxorubicin (ADM)-resistant THP1 cells and ADM sensitive cells and demonstrated that 49 circRNAs were differentially expressed (35 up- and 14 down-regulated) in doxorubicin (ADM)-resistant THP1 cells compared to ADM sensitive cells [57]. CircPAN3, which was upregulated in resistant cells, was selected for further experiments because of the known role of the parent gene (*PAN3*) in leukemogenesis. CircPAN3 was upregulated in relapsed/refractory compared to chemosensitive AML patients, though the parent gene expression did not follow a similar pattern. In cell lines, circPAN3 down-regulation was associated with restoring doxorubicin sensitivity, and in contrast, up-regulation was associated with doxorubicin refractoriness. The circPAN3-miR-153-5p/miR-183-5p-XIAP (X-linked inhibitor of apoptosis protein) axis was proposed as a mediator of drug resistance. Also, other investigators established circPAN3-mediated autophagy regulation via the AMPK/mTOR pathway as a resistance mechanism in AML cell lines [58]. Similarly, circ-ANXA2 expression was shown to be upregulated in AML patients [59]. Circ-ANXA2 expression correlated with poor-risk phenotype in AML patients, although multivariable analysis was not performed to discern the independent effects of circ-ANXA2 expression on treatment outcomes. In vitro, circ-ANXA2 knockdown (KD) was associated with increased apoptosis, reduced proliferation of AML cells, and increased chemosensitivity to cytarabine and daunorubicin. It was proposed that circ-ANXA2 possibly worked as a microRNA sponge to dampen the activity of miR-23a-5p and miR-503-3p (as levels of both were increased in circ-ANXA2-KD models). Both miRs are known to increase chemosensitivity [60, 61]. *ANXA2* (Annexin A2) expression was not evaluated in this study, but it was proposed that Circ-ANXA2 might enhance transcription of annexin A2, which has a well-established role in leukemogenesis [62, 63].

### Profiling circRNA in AML: experimental methods and computational tools

Structural uniqueness of circRNA can be challenging to study these molecules and it is one of the reasons for being less investigated than other components of the transcriptome. Sequencing of ribosomal RNA (rRNA) depleted total RNA was traditionally used in earlier studies [64]. Though comparative analysis of both linear and circRNAs simultaneously has obvious potential to provide insights to disease pathogenesis, there are technical challenges involved with this. For instance, it can be challenging to choose the level of sequencing depth and dependence on bioinformatics algorithms to identify circRNAs. More commonly used microarray analysis has less reliance on bioinformatics but most published studies have used “RNase R” based treatment for depletion of linear RNAs and hence comparative expression studies are not feasible. To study a specific circRNA of interest, many back-splicing junction (BSJ) locus-specific methods are available, e.g., northern blotting (less popular because of the need for large quantities of RNA and being labor intensive) and reverse transcriptase and quantitative polymerase chain reaction (RT-qPCR, frequently used in circRNA studies). More sensitive but expensive methods including droplet dense polymerase chain recation (ddPCR) and the nCounter platform from NanoString Technologies may provide opportunities for further research [65, 66]. Both these methods can digitally quantify circRNA. In situ hybridization using an oligonucleotide probe that spans the BSJ can identify the circRNA locus within the cell. Various databases have been developed with the help of data obtained from these experimental methods [67]. These databases are commonly used for circRNA identification, quantification, annotation, and network identification to reveal target and downstream signaling. Since the majority of these tools utilize advanced computer programming software, specific training is an absolute requirement.

### Limitations of current literature

The precise mechanism of regulation of circRNA expression in AML is not well studied. Except for fusion circRNAs, it is unclear if dysregulated circRNA expression is a primary event in leukemogenesis or an epiphenomenon. Also, most studies have used bone marrow samples, and only a few had peripheral blood samples. Correlative studies between bone marrow samples and peripheral blood are also lacking.

There is very limited knowledge about the metabolism and transport of circRNA within and outside the cell. It is known that surplus circRNAs are transported out of the cell in exosomes [68]. Although of great interest because of its well elucidated role in multiple other cancers [69], exosomal circRNA has not been studied in AML. Application of exosomal circRNA in modulating bone marrow microenvironment and extramedullary infiltration of leukemia cells can be an attractive area to study.

Relative expression of circRNA and the corresponding parent gene varies and thus may not be regulated in a uniform fashion across the entire genome. In a recent study, the expression of most circRNAs correlated with the expression of the parent gene in both AML patients and healthy controls, but there are instances of discordant expression [54], and also occasional relevant differences in the expression patterns between AML patients and healthy controls. As an example of the latter, circFLT3 expression correlates with parent gene expression only in healthy samples but not in AML patients. Many studies in our review did not examine the circRNA and its parent gene expression simultaneously. Whether any observed effect is from the circRNA or parent gene expression is also not evaluated in most studies, e.g., circ-VIM is associated with shorter overall survival in AML, but the impact of parent VIM gene expression is not studied. However, both followed a similar expression pattern [32].

Although KD circRNA studies represent a potential opportunity to assess the impact of circRNA expression on that of the parent gene, most studies reviewed herein did not evaluate the circRNA-KD effect on the parent gene expression [38, 41, 44, 48, 49, 52, 53, 59]. Further, much work remains to be done in the area of modeling the interplay of intracellular effects resulting from dysregulation of multiple circRNAs occurring simultaneously.

## Conclusion

AML is a challenging, biologically heterogenous disease with an unmet need for novel diagnostic, therapeutic, and prognostic markers. In this review, we summarized the biological and clinical significance of circRNA in AML. The role ncRNAs, particularly circRNAs, in each of these clinical facets, as well as of leukemogenesis in general is an evolving horizon.

## Data Availability

Not applicable.

## Abbreviations

AML: Acute myeloid leukemia
CircRNA: Circular RNA
ncRNA: non-coding RNA
mRNA: Messenger RNA
RBP: Ribosomal binding protein
miRNA: MicroRNA
RT-qPCR: Reverse transcriptase and quantitative polymerase chain reaction
WBC: White blood cells
FAB: French-American-British
ALL: Acute lymphoblastic lymphoma
MRD: Minimal residual disease
HDGF: heparin-binding nuclear growth factor
RNF13: RING finger protein 13
PTBP1: Polypyrimidine tract-binding protein 1
AUC of ROC: Area under the receiver-operating characteristic curve
CN-AML: Cytogenetically normal AML
KD: Knockdown
EMI: Extramedullary infiltrating
BM: Bone marrow
ADM: Doxorubicin
rRNA: Ribosomal RNA
BSJ: Back splicing junction
ddPCR: Droplet dense polymerase chain reaction
